# Microfungi, Mycotoxins and Heavy Metals in the Mass Rearing of *Tenebrio molitor* L. Larvae

**DOI:** 10.3390/insects17080866

**Published:** 2026-08-20

**Authors:** René Rehorska, Valentin Kraus, Nicole Bodrik, Florian Lehnhardt, Mathias Hutzler, Martina Gastl, David Renault, Simon Berner

**Affiliations:** 1 Institute Applied Production Sciences, University of Applied Sciences FH JOANNEUM, Alte Poststraße 149, 8020 Graz, Austria; 2 Research Center Weihenstephan for Brewing and Food Quality, Technical University of Munich, 85354 Freising, Germany; 3 EcoBio (Ecosystèmes, Biodiversité, Évolution), UMR 6553, CNRS-Université de Rennes 1, 263 Avenue du Général Leclerc, 35042 Rennes Cedex, France; david.renault@univ-rennes.fr

**Keywords:** *Tenebrio molitor*, microfungi, mycotoxins, heavy metals, chromium

## Abstract

Since *Tenebrio molitor* L. was approved by the EU as novel food in 2021, food safety is paramount for both chemically and microbiologically safe products. Since *T. molitor* larvae are reared on substrates mainly based on cereals, mycotoxins and heavy metals may represent a serious risk. However, analyses of mycotoxins and heavy metals are complex and cost-intensive procedures, which may not be affordable for small-scale farmers on a regular basis. To provide a preliminary risk assessment and to add further insights to existing knowledge, this study used a rapid screening method based on MALDI-TOF to identify potentially mycotoxin-producing microfungi. The colony-forming units (CFU) were determined, and the isolated species identified. In addition, a_w_-values of substrates and meal (four samples, nine repeats each) were determined to assess mycotoxin-related risks. Eight mycobionts were identified, including three potential mycotoxin producers: *Penicillium citrinum*, *Aspergillus flavus*, and *Aspergillus brasiliensis*. Very low CFU counts (<3 × 10^3^ CFU/g) and a_w_-values (0.35–0.59) indicate minor risks. LC-MS/MS mycotoxin analysis of nine samples (varying in source and time point) confirmed concentrations below the limit of detection (LoD): 0.50 µg/kg for aflatoxins B1/B2/G1/G2; 50 µg/kg for DON/FuB1/FuB2; 20 µg/kg for HT2/T2; 150 µg/kg for NIV; 1 µg/kg for OTA; and 5 µg/kg for ZEA. Heavy metal analysis (ICP-OES/MS) of three substrates and insect meal showed that all six metals (Cd, Pb, Hg, Sb, As, Cr) were below the LoD (0.2 mg/kg), except for Cr in malt residual pellets (0.4 mg/kg).

## Simple Summary

This study investigates the safety of the mass rearing procedure of *Tenebrio molitor* mass rearing and its products by investigating microfungal contamination and heavy metal accumulation in a preliminary risk assessment. While mycotoxin-producing fungi were detected on substrates and insect meal, their low abundance and the dry rearing conditions minimized mycotoxin risk. Analyses confirmed that mycotoxin and heavy metal concentrations were below detection limits, except for chromium in malt residual pellets. This preliminary risk assessment provides an initial, yet methodologically comprehensive, evaluation of potential contamination risks in *Tenebrio molitor* rearing.

## 1. Introduction

### 1.1. Background

The approval of insects as novel foods in the European Union was a lengthy and scientifically rigorous process, supported by numerous investigations into the chemical and microbiological safety of insect-based foods [1]. This applies equally to the yellow mealworm beetle *Tenebrio molitor*: this species can be considered one of the most thoroughly researched insects for human consumption. The data on its chemical and microbiological safety are correspondingly extensive and include the substrates used to rear T. molitor larvae. Although the microbiological contamination of the larvae is comparable to that of commercially available meat, the use of agricultural by-products as feed introduces potential risks, such as heavy metal accumulation and mycotoxins [2,3,4]. Bacterial communities associated with *T. molitor* include members of *Enterobacteriaceae* (*Enterobacter* spp., *Klebsiella* spp.), lactic acid bacteria (*Enterococcus* spp., *Lactococcus* spp.), and *Staphylococcus*, *Bacillus*, *Pseudomonas* and *Clostridium* species [5]. With respect to the heavy metal concentration in by-products used as substrates, Truzzi et al. (2019) found that organic wheatmeal showed the highest concentrations of Cd and Pb, whereas As and Ni were predominant in olive pomace [4]. At the time, in both cases the concentrations were considered low enough to classify these by-products as safe animal feed for *T. molitor* [4]. However, considering current regulations, this assessment should be treated with the utmost caution and reconsidered. Considering mycotoxins such as deoxynivalenol (DON), which predominantly occurs in barley and wheat, there is also evidence that uptake by the larvae, even when fed with contaminated substrates, is not high enough to pose a substantial health risk [6,7]. There have been very similar findings on other mycotoxins produced by the genus Fusarium, such as zearalenone (ZEN) [8]. However, most studies on *Tenebrio molitor* focus on *Fusarium* mycotoxins, and little is known about non-*Fusarium* fungal species and their potential mycotoxins in insect rearing. This gap is particularly relevant as substrates may harbor other mycotoxin-producing fungi, such as *Aspergillus* or *Penicillium*, which remain insufficiently characterized in this context [7].

### 1.2. Knowledge Gap

This naturally raises the question of why microfungi should be investigated at all in the production of *T. molitor* if they, or their metabolic products, do not pose any significant risk. There are two answers to this question. Firstly, although there is comprehensive scientific literature available on mycotoxins occurring in mass rearing of *T. molitor*, there is very little information on microfungi species associated with these insects and their breeding environment, which is kept under very specific conditions. Naturally, the focus here has been on relevant mycotoxins themselves in the context of food safety, rather than their fungal producers. Secondly, most studies here deal with *Fusarium*-toxins, which are certainly relevant for cereal-based substrates. However, this does not mean that other mycotoxins cannot also be present in feed for insects—and in insects themselves, which may be produced by culprits that are not as obvious as *Fusarium graminearum* or *Fusarium fujikuroi* [9]. Considering feed for *T. molitor*, the majority of used substrates, if pre-formulated or derived directly from agricultural by-products, consists at least partially of plant-based nutrients [10]. In general, the phylloplane and rhizosphere of plants is inhabited by a large variety of different microorganisms, including mycobionts [11]. Cultivated crops such as *Glycine max* (L.) Merr. (soybean), *Triticum aestivum* L. (wheat) and *Hordeum vulgare* (barley) provide ecological niches to specific mycobionts due to their high protein and starch content [12,13]. The infection of protein plants and starchy crops with phytopathogenic fungi will become noticeable before harvest, depending on the severity of the infection, but it will be monitored, in any case, very thoroughly by visual and analytical methods [14]. Hence, it is comprehensible that typical phytopathogenic fungi are primarily mentioned in the context of *T. molitor* substrates. A thorough analysis shows that there are more fungal species involved in potential contaminations, which can be already present in the original plant, or can be transferred to the product during the harvest or the down-stream processing in mills, or may be present as airborne and environmental contaminants in the insect-breeding facilities, or may even originate from the insects themselves [15,16]. Finally, the following speculation should be mentioned as to why microfungi should be investigated more comprehensively in the context of insect breeding. The legal requirements and regulations for ensuring the safety of insect-based foods are becoming stricter and more extensive, rather than more lenient. The threshold values for mycotoxins and heavy metals are likely to fall rather than rise [17,18].

### 1.3. Scope of the Study

Employing a wide range of analytical methods, this study investigates mycotoxin and heavy metal contamination in *Tenebrio molitor* larval rearing to provide a preliminary risk assessment and identify potential contamination sources. The presented data cover substrates and insect meal from both university research and commercial production. However, it is important to note that this study does not constitute a comprehensive or definitive risk assessment of mycotoxin or heavy metal contamination in *T. molitor* substrates or larval-derived products. Fungal species and heavy metals that occur in two different substrates (wheat bran and a professional feed-formulation applied in a mass rearing facility), in the climate-controlled insect-breeder and in the meal (provided by a mass rearing facility) from insect larvae of *T. molitor*, were investigated.

A major part of the analyzed samples originated from the insect rearing site of the FH JOANNEUM, which has been in continuous operation since 2019. The identified contaminations may be regarded as comparable to contaminations occurring in small-scale insect rearing enterprises: the rearing conditions and the composition of the feed mixtures do not differ significantly across most *T. molitor* rearing facilities in Central Europe. The substrates used often contain a high proportion of wheat bran and are dry substrates with low a_w_-values. The growth conditions are at temperatures above 25 °C and a relative humidity of well below 80% [19,20].

## 2. Materials and Methods

### 2.1. Air Sampling in the Insect-Breeder

The experimental insect-breeding facility of the FH JOANNEUM University of Applied Sciences Graz (8020 Graz, Austria) was sampled to investigate microfungi associated with the breeding environment of insects. The population of *Tenebrio molitor* at this facility is bred in two WTB-Binder climate chambers (KBF-S, Binder GmbH, 78532 Tuttlingen, Germany) since 2019. The sampled climate chamber contained, at the time of sampling, 10 boxes of *T. molitor* larvae, pupae and beetles, which are kept constantly at 26 °C and 60% relative humidity (RH) on wheat bran standard feeding substrate. Air sampling was conducted on 7 plates of Sabouraud Gentamicin Chloramphenicol 2 (SGC2) agar, derived from bioMérieux Austria GmbH (1100 Vienna, Austria).

SGC2 is a selective culture medium for the isolation of yeasts and molds. Two air samples were collected on each of the drawers of the climate chamber and one on its ground floor respectively for 24 h. The seven SGC2 agar plates were positioned as follows: one on the chamber floor (0 mm) and two on each of the three shelves above at heights of 150 mm, 300 mm, and 450 mm. Plates were exposed for 24 h and incubated at 30 °C for 4 days in a Heratherm IGS 750 incubator (Thermo Fisher Scientific Inc., 02454 Waltham, MA, USA). This method aligns with standard passive air sampling protocols using open Petri dishes, according to Pasquarella et al. (2012) [21].

### 2.2. Sampling During Rearing Cycles of T. molitor Larvae

**Figure 1:**
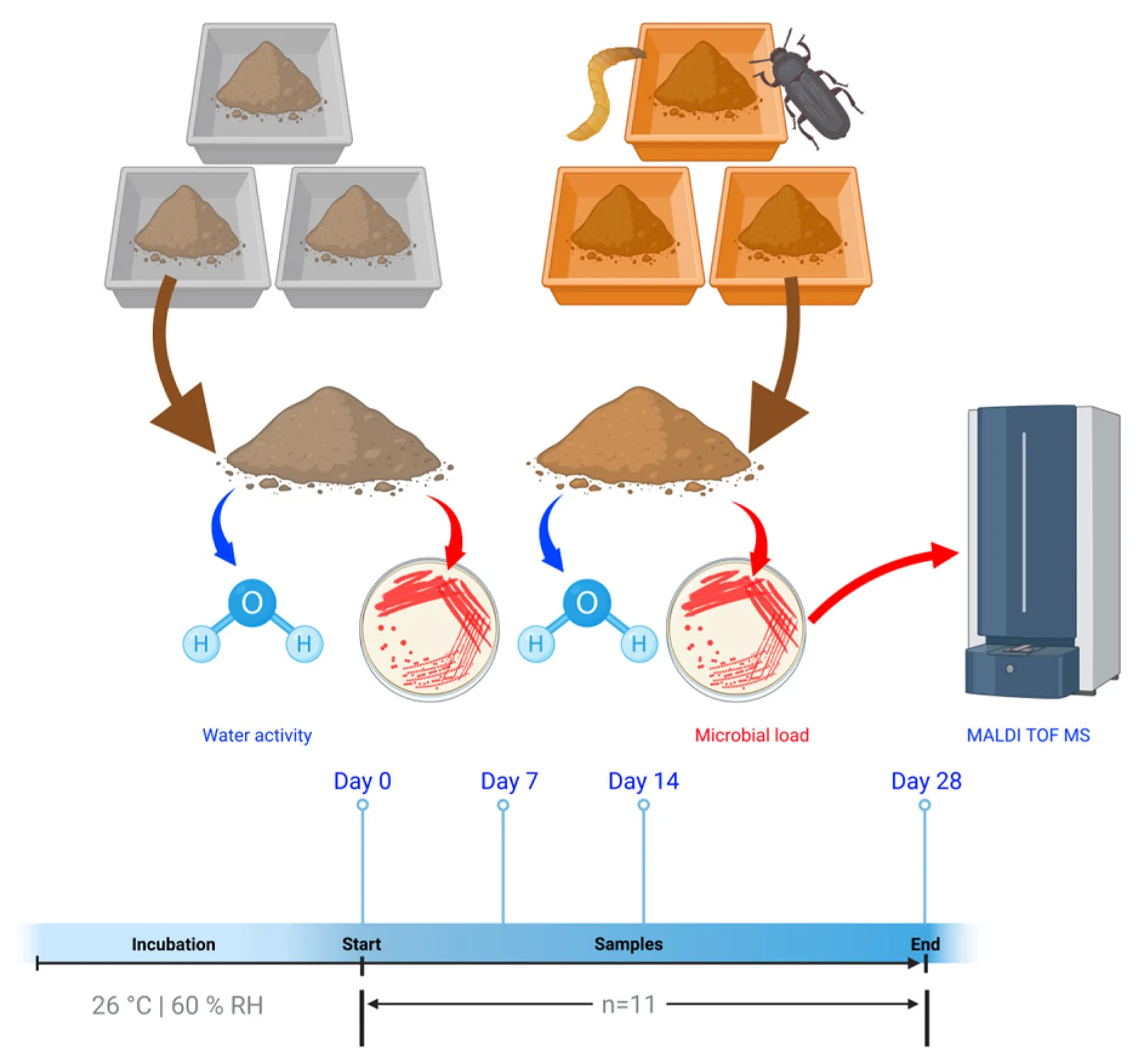
Experimental design to determine microfungal load during active rearing cycles of *T. molitor*; three boxes with wheat bran harbored *T. molitor* larvae, and three boxes were prepared with wheat bran only as controls. The experiment was repeated three times. Created in BioRender. Rehorska, R. (2026) https://BioRender.com/xphfxs0 (accessed on 1 July 2026).

To monitor microfungal growth occurring during active rearing cycles of *T. molitor*, three polypropylene boxes (15 cm × 23 cm × 5 cm) prepared with 200 g of wheat bran from Auer Mühle GmbH (4451 Garsten, Austria) were populated with approximately 0.50 g of larvae each. This corresponds to 19 individuals per box and to a stocking density of 1 larva per 45 cm^3^. The larvae were 4 weeks old when the boxes were stocked, with an average larval weight of 27 mg. The *T. molitor* population used in this study originated from larvae that were derived in 2019 from Prime Insect (9462 Bad St. Leonhard im Lavanttal, Austria). Three boxes were prepared with 200 g of wheat bran only as controls. The temperature during the rearing of the larvae was kept constant at 26 °C and 60% relative humidity (RH). At the start of the experiment (day 0), samples from both inhabited substrate and control substrate were taken. To determine which effect *T. molitor* and its frass has on the a_w_-value of wheat bran, no additional sources of water such as carrots were added. The a_w_-value was measured in triplicates for substrates from each box (6 boxes per time point (3 with larvae, 3 controls) totaling 72 measurements). Furthermore, an SGC2 plate was inoculated for each box. For this purpose, 1 g of sample was mixed with 29 mL of sterile buffered peptone water (BPW, derived from bioMérieux Austria GmbH) in sterile 50 mL falcon tubes and vortexed thoroughly. The sample then was put on a shaker plate for 30 min, followed by centrifugation (9600 rpm, 5 min, 25 °C). Afterwards, 1000 µL of the solution were transferred to a SGC2-plate and spread evenly using a Drigalski spatula. The plates then were incubated at 30 °C for four days and the colony-forming units were determined. Isolated colonies were identified at TUM Research Center Weihenstephan for Brewing and Food Quality (TUM BLQ, 85354 Freising, Germany) by MALDI-TOF (method designation: Maldi-TOF, SAA 92540, 2018-08). The inhabited boxes and the controls were sampled in the same manner on day 7, 14 and 28. Samples for mycotoxin analyses were taken from the control before and after seven days of incubation (wheat bran D0; D7), immediately after the stocking with the larvae (wheat bran larvae D0) and after seven days of incubation in the climate chamber (wheat bran larvae D7). The experiment was repeated three times, and the samples for the mycotoxin analyses were pooled. The overall experimental design is shown in Figure 1.

### 2.3. Microfungal Load of Formulated Substrate and Larvae Meal Derived from Industry

In addition, to derive comparable results for industrial breeding, the microfungal load and the a_w_-value of a commercially used substrate formulation for the mass rearing of *T. molitor* larvae, derived from a mass rearing facility (Ÿnsect Ltd. (91058 Évry, France)), were investigated. In accordance with standard industrial practice, *T. molitor* larvae are dried using convection drying at a temperature of 60 °C [22]. To put the results of all investigations into context with the final product for further processing, dried larvae meal from *T. molitor* from Ÿnsect Ltd. was analyzed in the same manner. In detail, the number of independent replicates was as follows. Mycotoxin analysis (HPLC-MS/MS): 9 samples (W. bran control (D0, D7), W. bran larvae (D0, D1, D7), Larvae meal (Ynsect, FHJ), substrate formula (Ynsect) and malt residual pellets (MRP)) were tested, each analyzed in double determination.

Heavy metal analysis (ICP-OES/MS): 4 distinct substrates (malt residual pellets, wheat bran, commercial diet, insect meal) were analyzed, each in double determination.

a_w_-measurements (ISO 18787): 3 random samples were taken from the formulated substrate as well as from the larvae meal [23]. Each were measured in triplicates resulting in 18 measurements total.

Sample preparation for the microfungal analysis was conducted identically as described above.

### 2.4. Determination of Water Activity (a_w_-Value)

Since microbial growth is predominantly determined by water activity, the a_w_-value is essential to assess the potential risk of microbial contamination before even determining the specific microbial load. The a_w_-values of substrate and meal (as described in Section 2.2 and Section 2.3) were analyzed using the Labmaster a_w_-neo (Novasina AG, 8853 Lachen, Switzerland) following the ISO 18787 method using 2.00 g of sample per analysis. This device employs a temperature-controlled measurement chamber (0–60 °C, ±0.1 °C precision). The measurements were according to the ISO 18787:2017 standard, which defines critical parameters for water activity testing, including temperature control at 25 ± 1 °C and the following stability criteria: the final measurement point is determined upon reaching a plateau with a maximum amplitude variation of 0.0003 a_w_ across three consecutive measurements, each stable for 1 min [23]. The ISO mode is pre-configured on the LabMaster-a_w_ neo to ensure compliance with international testing requirements for food safety and quality control.

### 2.5. Species Identification by MALDI-TOF

The rapid and inexpensive identification method which was used to screen the samples, based on MALDI-TOF, is a standardized method applied by the TUM BLQ, to identify microorganisms isolated from foods and beverages (method designation: MALDI-TOF, SAA 92540, 2018-08). This method proved to be perfectly suited to identify the isolated microfungi rapidly. For MALDI-TOF identification, material was taken from each marked colony on the respective agar plates. The spectra were generated using the Microflex™ device and compared through the Biotyper software Version MBT Compass 4.1 from Bruker^®^ (01821 Billerica, MA, USA) with the database available at TUM BLQ. This comparison provided a report on results, with a score assigned by the software for each comparison. Score levels ≥2 represented an identification categorized as “most likely,” score levels between 1.7 and 1.99 represented an identification categorized as “likely,” and score levels ≤1.69 represented an identification categorized as “no identification possible.” For mold colonies, subsequent analysis was performed using the extraction method according to the manufacturer’s instructions. All identification score levels of this study were above 1.9.

### 2.6. Mycotoxin Analysis with LC-MS/MS

To align the results of the MALDI-TOF analysis with the actual mycotoxin content, a mycotoxin analysis of the same substrate and insect meal was performed using LC-MS/MS. This method was also used to analyze malt residual pellets (MRP), a by-product of the malting industry. These pellets, derived from dried and pressed seedlings, were obtained from STAMAG Stadlauer Malzfabrik GesmbH, 8020 Graz, Austria. MRPs are used as promising mono-substrates for a second population of *T. molitor* at the FH JOANNEUM breeding site and may be a promising alternative to wheat bran. The analysis of mycotoxins was performed on a LC system (1290 Infinity, Agilent, 76337 Waldbronn, Germany) coupled to a triple quad tandem mass spectrometer (G6470A, Agilent, 76337 Waldbronn, Germany) according to DIN EN 17194:2020 [24]. The following parameters were analyzed: aflatoxin B1 (AFB1), aflatoxin B2 (AFB2), aflatoxin G1 (AFG1), aflatoxin G2 (AFG2), deoxynivalenol (DON), fumonisin B1 (FuB1), fumonisin B2 (FuB2), HT-2 toxin (HT2), nivalenol (NIV), ochratoxin A (OTA), T-2 toxin (T2), and zearalenone (ZEA). The mycotoxin analysis was limited to day 7 because the measured water activity in all substrates and insect meal ranged from 0.35 to 0.59 a_w_, which is well below the minimum a_w_ threshold of ≥0.80 required for mycotoxin production by common toxigenic fungi such as *Aspergillus* spp. and *Penicillium* spp. [25]. Since mycotoxin synthesis is strongly inhibited at a_w_-values below 0.80, no significant toxin accumulation was expected beyond this point. Additionally, repeated sampling for mycotoxin analysis would have required the removal of substantial amounts of substrate, which could negatively impact larval development and survival. Nevertheless, fungal growth was monitored until day 28 to confirm that microbial contamination remained low under the stable, dry rearing conditions (60% RH, 26 °C), ensuring that no unexpected shifts in a_w_ or microbial load occurred during the experiment.

### 2.7. Multi-Element Analyses

To complement the analytical characterization of the samples, a multi-element analysis was performed to quantify major and minor elemental composition. The measurements were conducted using an inductively coupled plasma optical emission spectrometer (ICP-OES; Optima 8300, PerkinElmer, 06484 Shelton, CT, USA) in accordance with DIN EN ISO 11885:2009-09 [26]. The method enabled the determination of elements such as aluminum (Al), boron (B), barium (Ba), calcium (Ca), copper (Cu), iron (Fe), potassium (K), magnesium (Mg), manganese (Mn), sodium (Na), phosphorous (P), sulfur (S), silicon (Si), strontium (Sr) and zinc (Zn).

In addition, the samples were analyzed for trace elements and heavy metal contaminants using inductively coupled plasma mass spectrometry (ICP-MS; NexION 2000, PerkinElmer, 06484 Shelton, CT, USA) following DIN EN ISO 17294-2:2024-12 [27]. This method was applied to quantify elements present at trace levels, including arsenic (As), cadmium (Cd), chromium (Cr), cobalt (Co), nickel (Ni), lead (Pb), mercury (Hg), antimony (Sb), selenium (Se), uranium (U) and vanadium (V).

## 3. Results

Overall, the total colony-forming units of molds present on selective media were at a low level (<10^3^ CFU/g), which is consistent with the results of comparable literature [3,28].

When examining the wheat bran substrate in which the mealworms were reared in-house, as well as the wheat bran control in-house, *Penicillium* was the most prevalent genus identified. In contrast, with the formulated substrate used for mass rearing and the processed insect meal, the most abundant genus was *Aspergillus*.

The concentrations of all detected mycotoxins in wheat bran and larvae meal were below the limit of detection.

### 3.1. Mycobionta Isolated from T. molitor Substrates and Insect Meal

Table 1 lists the microfungal species identified via MALDI-TOF, including three potential mycotoxin producers (*Penicillium citrinum*, *Aspergillus flavus*, *A. brasiliensis*). However, colony-forming unit counts were very low (<3 × 10^3^ CFU/g), which are below the fungal contamination levels reported for substrates used in the rearing of *T. molitor*, with reported CFUs of up to 6.4 × 10^3^ CFU/g [3]. Nonetheless, the use of pooled samples limits the detection of localized contamination hotspots, and the qualitative nature of MALDI-TOF identification does not allow for quantitative risk assessment. Four different species of *Aspergillus* were isolated from wheat bran, as it is shown in Table 1. Among these species, which were identified by MALDI-TOF, two are known to produce mycotoxins: *A. flavus*, which can form aflatoxins, and *A. niger*, which forms ochratoxin A. In addition, *Penicillium citrinum*, which produces citrinin, was identified as another potential mycotoxin-forming mold.

*Rhizopus oryzae* is a saprophytic filamentous fungus which belongs to the Mucoraceae, and which is used in biotechnology, especially in the production of enzymes such as cellulases, lipases and proteases [29,30]. *Didymella pedeiae* is a phytopathogen and a potential human pathogen ascomycetous fungi, which at least in one case was reported to cause fungal rhinosinusitis in an immunosuppressed patient [31].

### 3.2. a_w_-Values over the Course of the Breeding Experiment

**Figure 2:**
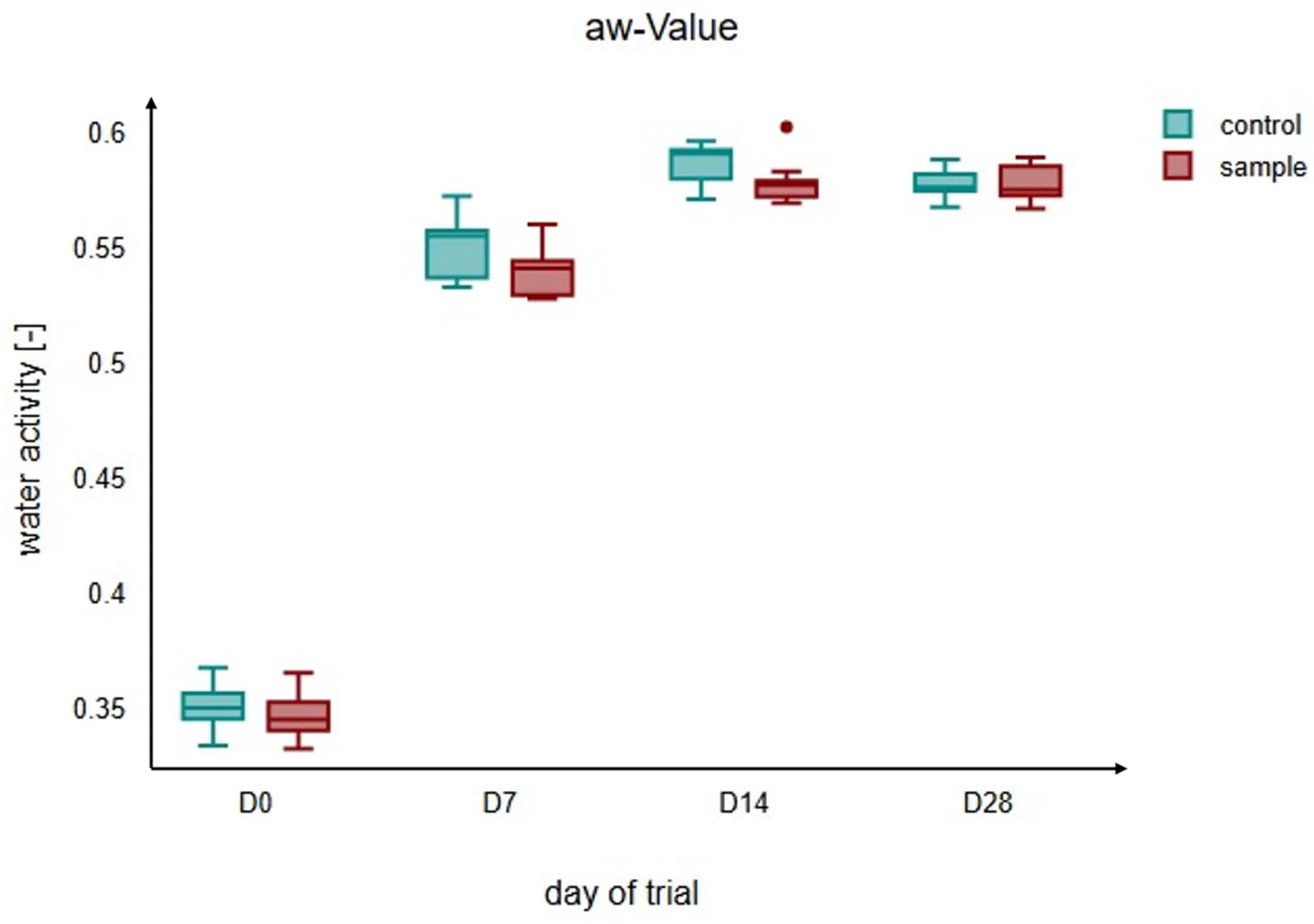
a_w_-values of wheat bran (control) and wheat bran inhabited by *T. molitor* larvae over the course of 28 days. Shown are means and standard deviations. The insects were reared at 26 °C and 60% of relative humidity.

Figure 2 shows that a_w_-values in all substrates and insect meal ranged from 0.35 to 0.59, remaining stable over the 28-day observation period. These values are well below the minimum threshold of ≥0.80 required for mycotoxin production by common toxigenic fungi such as *Aspergillus* and *Penicillium* spp. [32]. However, a_w_-measurements were taken from a limited number of samples under controlled laboratory conditions, which may not fully represent industrial-scale variability. The a_w_-values in both control and samples as described in Figure 2, increased over the course of the incubation, but stabilized after the first week at approximately 0.60, correlating with the humidity conditions (60%) in the breeding chamber. In comparison the substrate and meal provided by Ÿnsect Ltd. had values of 0.55 and 0.39 respectively. This indicates unsuitable conditions for most problematic microorganisms, including molds and other microfungi [33]. However, it should be noted that these results, which are based on a limited number of samples, cannot be regarded as universally applicable.

### 3.3. Mycotoxin Analyses

The concentrations of all mycotoxins in the wheat bran and larvae meal samples, given in Table 2, were below the limit of the detection of the applied method (DIN EN 17194:2020, modified). The only sample that showed mycotoxin concentrations above the limit of detection was the malt residual pellets derived from a local malting plant, concerning the aflatoxin variants that were analyzed. Malt residual pellets can be used as monosubstrate in insect feed, so these results may be of relevance to insect mass rearing, where buying and storing MRP as bulk material is considered. Comparable studies report Aflatoxin B1 concentrations in MRP of up to 1.2 µg/kg [34]. The results in the present study are consistent with the low a_w_-values (Figure 2) and minimal fungal contamination. EU Directive 2002/32/EC sets a legal maximum of 0.02 mg/kg for aflatoxin B1 in feed (0.005 mg/kg for dairy ruminants), while other mycotoxins such as deoxynivalenol, zearalenone, or ochratoxin A only have recommended values under Commission Recommendation 2006/576/EC [35,36]. The test results shown in Table 2 are below the EU limits. As the limit of detection does not guarantee absolute absence, local contamination or temporary fluctuations cannot be ruled out. However, the absence of detectable mycotoxins does not guarantee their absence, as levels may have been below the analytical sensitivity or temporally variable.

### 3.4. Multi-Element Analyses

Table 3 and Table 4 present heavy metal concentrations in substrates and insect meal, respectively. All six analyzed metals (Cd, Pb, Hg, Sb, As, Cr) were below the limit of detection (0.2 mg/kg), except for chromium in malt residual pellets (0.4 mg/kg). These levels are low compared to literature data from van der Fels-Klerx et al. (2016), who demonstrated that *Tenebrio molitor* larvae can accumulate heavy metals such as cadmium, lead, and arsenic from contaminated substrates, with concentrations in larvae exceeding EC maximum limits for feed materials under certain conditions [37]. However, the detection limit of 0.2 mg/kg in the present study may mask lower contamination levels, and metal accumulation in *T. molitor* is highly dependent on the substrate. In addition, direct comparisons are limited by differences in substrate composition and analytical methods. Thus, while the results indicate low heavy metal content, the absence of detectable levels does not guarantee safety, particularly if substrates with higher background contamination are used.

## 4. Discussion

### 4.1. Mycotoxins and Water Activity

While all other samples (wheat bran controls, wheat bran with larvae, commercial substrate, and larvae meal from Ÿnsect and FHJ) showed mycotoxin concentrations below the limit of detection, only malt residual pellets (MRP) exhibited detectable levels of aflatoxin B1 (0.97 µg/kg), B2 (0.56 µg/kg), G1 (4.36 µg/kg), G2 (7.18 µg/kg), and HT-2 (57.27 µg/kg). These substrate-specific differences are attributable to the origin and pretreatment of MRP as a by-product of the malting industry. The findings apply exclusively to the samples tested here under the applied analytical conditions (LC-MS/MS with LoD of 0.50 µg/kg for aflatoxins), rearing conditions (26 °C, 60% RH), and experimental parameters, and cannot be generalized to other substrates, production facilities, or storage conditions. Regarding the mycotoxin contamination of *T. molitor* products reared on plant-based substrates in the introduced laboratory setting, the preliminary risk assessment indicated no substantial risks of contamination. Low a_w_-values, which are not significantly increasing during growth of the insects on the substrates prevent microbiological growth—and that of many potentially harmful fungi. It was also found that the mycotoxin concentrations in the plant-based substrates used were below the limit of detection. The only exception was the aforementioned aflatoxins. As both the wheat bran and the MRP used were animal feed from commercial producers, it could be assumed that the relevant statutory limit values would not be exceeded [36]. Nevertheless, it must be noted that the present study was only conducted for 28 days and at a relatively low air humidity of 60% RH. This is within the recommended range for larval growth, but it may not reflect the typical breeder’s conditions, nor may it represent the average storage conditions of the substrates before they are stocked with larvae. The optimal rearing range for *T. molitor* is reported to be between 50 and 75% RH [38]. Thus, a higher water content, which may positively increase larval growth, may also promote the growth of micro fungi and the production of certain mycotoxins. On the other hand, high temperatures and drought may increase the production of specific mycotoxins, such as fumonisins (FUM) [39]. However, there is one caveat to our findings: xerophilic and osmophilic fungi may not have been detected due to the rather generalized medium for yeasts and fungi used in this work. These fungal species are adapted to arid environments and those with high salt concentrations, and can be isolated on DG-18 (Dichloran-Glycerin-Chloramphenicol)-Agar [40,41]. Wheat bran can definitely be regarded as a very dry substrate according to the measured a_w_-values of approximately 0.6. According to Coleine et al. (2022), *Aspergillus penicillioides*, *Xeromyces bisporus* and *Zygosaccharomyces rouxii* are fungal species occurring at these low levels of available moisture [41]. According to Stevenson et al. (2022), *A. penicillioides* is remarkably tolerant to extreme conditions and shows active cell division at water activity levels outside the currently understood window for life [42]. *X. bisporus* can cause food spoilage, but to form spores it needs higher levels of water activity [43,44]. *Z. rouxii* is a feared beverage spoilage organism in wine and juice production, but it is also intentionally used in the production of soy sauce [45,46]. These three extremophilic microfungi may still cause spoilage of plant-based mono-substrates with very low water activity in insect rearing and should be given greater attention in future studies regarding any mycotoxins that may be produced.

### 4.2. Heavy Metals

Regarding the concentrations of the heavy metals in the analyzed samples, the results are similar and are basically in accordance with Truzzi et al. (2019) [4]. The majority of heavy metals that can be considered problematic were below the limit of detection. The only exception, chromium, was detected at a very low concentration in MRP with 0.4 mg/kg. In comparison to that, Mateos et al. (2002) analyzed breakfast cereals in Spain for their chromium content and found average concentrations of 0.23 mg/kg [47]. The chemistry of chromium regarding its biological functions as an essential element and its toxic, carcinogenic form Cr(VI) is complex [48]. While Cr(III) is biologically significant as an essential trace element and is found in numerous foods and agricultural products, Cr(VI) is a human carcinogen that poses serious risks to human health. For this reason, when determining the total chromium content, it is essential to note that only a further, detailed analytical and toxicological investigation of the chromium species present can provide certainty in this regard. Hence, it should not be just stated as beneficial regarding the results of this study. However, it can be concluded that the detected total concentration is comparable to those reported in similar studies on grain products. It is not possible to deduce a region-specific concentration of Cr directly from this, as the studies compared originate from Spain, while the cereal products examined in this article originate from Austria. This does not, of course, imply that the possibility of regional contamination can be ruled out. However, it may also be a case of general contamination, which may stem from the treatment and processing of seeds and cereals, regardless of region [47,49,50]. Currently, only few and partly contradictory studies exist on heavy metal accumulation in *T. molitor larvae* [4,37,51]. However, there is at least one study that points to a potentially harmful accumulation of heavy metals such as Zn, Cd, Pb and As in insects, in general [51]. Research regarding specifically chromium in *T. molitor* larvae has not yet been conducted to our knowledge. Thus there remains a significant knowledge gap in this area, requiring more in-depth and specific studies, especially since there is evidence for accumulation of other heavy metals during the rearing process.

## 5. Conclusions

This study demonstrated, to a limited extent, that when using recommended substrates and moisture regimes in the cultivation of *T. molitor*, not only do very low a_w_-values prevail during the larval rearing phase in the incubator, but the CFUs of isolated and potentially mycotoxin-producing microfungi are also low. This is consistent with the results of the LC-MS/MS-based mycotoxin analyses of the substrate, substrate stocked with larvae, and larvae meal, which revealed concentrations predominantly below the limit of detection for a wide range of mycotoxins relevant to the animal feed sector. The findings regarding heavy metal concentrations are similar, at least based on the findings of this study and the substrates that were analyzed. Although this is merely a single study and the results must certainly be validated for industrial rearing and applications in insect farming, the findings of this preliminary risk assessment indicate that, at least with the specific raw materials used, the specific rearing conditions applied, and the specific detection methods employed, no exorbitantly high-risk factors relating to contamination with mycotoxins and heavy metals could be identified. These results by no means imply that these risks do not exist, and the findings must therefore, under no circumstances, be interpreted as universally applicable. Rather, far more extensive studies, including those conducted in commercial settings, would be required for validation, as there is considerable variability in the raw materials used, the rearing conditions and the processing methods. Future scientific investigations in commercial *T. molitor* facilities should focus on systematic substrate screening of incoming goods, and the continuous determination of a_w_-values and climate monitoring during storage. Periodic sampling should be performed throughout processing to detect contamination patterns, with final product analysis ensuring compliance with mycotoxin and heavy metal limits. This approach, logically aligned with critical control points, enables targeted risk assessment while acknowledging that findings are specific to the tested substrates and conditions.

## Figures and Tables

**Table 1:** Identified species of microfungi in two different substrates used to rear *T. molitor*: wheat bran and a special substrate formular used for commercial rearing of *T. molitor*.

Sample	Genus	Species	Mycotoxin
Insect meal Ÿnsect Ltd.	Aspergillus	*A. flavus*	Aflatoxins
Formulated substrate; Insect meal Ÿnsect Ltd.		*A. tritici*	
Formulated substrate; Insect meal Ÿnsect Ltd.		*A. montevidensis*	
Formulated substrate		*A. niger*	Ochratoxin A
Insect meal Ÿnsect Ltd.		*Aspergillus flavus* var. *oryzae*	
Formulated substrate	Penicillium	*P. citrinum*	Citrinin
Airborne fungi in climate camber		*P. cellarum*	
Airborne fungi in climate camber		*P. solitum*	
Wheat bran		*P. dipodomyicola*	Patulin
Wheat bran during rearing		*P. griseofulvum*	Griseofulvin
Wheat bran during rearing		*P. expansum*	Patulin
Formulated substrate	Rhizopus	*R. oryzae*	
Insect meal Ÿnsect Ltd.	Didymella	*D. pedeiae*	

**Table 2:** Concentrations of mycotoxins in Wheat bran (W. bran) control trials and Wheat bran stocked with larvae, in meal from larvae (insects bred at Ynsect and FHJ), in professional substrate formula from Ynsect, and in malt residual pellets usually sold as fertilizer or animal feed by malting companies. Concentrations are given in µg/kg of sample. The according limits of detection are given in the footer of the table.

Sample	AFB1 ^1^	AFB2 ^1^	AFG1 ^1^	AFG2 ^1^	DON ^2^	FuB1 ^2^	FuB2 ^2^	HT2 ^3^	NIV ^4^	OTA ^5^	T2 ^3^	ZEA ^6^
W. bran control D0	<0.50	<0.50	<0.50	<0.50	<50	<50	<50	<20	<150	<1	<20	<5
W. bran control D7	<0.50	<0.50	<0.50	<0.50	<50	<50	<50	<20	<150	<1	<20	<5
W. bran larvae D0	<0.50	<0.50	<0.50	<0.50	<50	<50	<50	<20	<150	<1	<20	<5
W. bran larvae D1	<0.50	<0.50	<0.50	<0.50	<50	<50	<50	<20	<150	<1	<20	<5
W. bran larvae D7	<0.50	<0.50	<0.50	<0.50	<50	<50	<50	<20	<150	<1	<20	<5
Larvae meal (Ynsect)	<0.50	<0.50	<0.50	<0.50	<50	<50	<50	<20	<150	<1	<20	<5
Larvae meal (FHJ)	<0.50	<0.50	<0.50	<0.50	<50	<50	<50	<20	<150	<1	<20	<5
Substrate Formula (Ynsect)	<0.50	<0.50	<0.50	<0.50	<50	<50	<50	<20	<150	<1	<20	<5
MRP	0.97	0.56	4.36	7.18	<50	<50	<50	57.27	<150	<1	<20	<5

^1^ LoD 0.50 µg/kg; ^2^ LoD 50 µg/kg; ^3^ LoD 20 µg/kg; ^4^ LoD 150 µg/kg; ^5^ LoD 1 µg/kg; ^6^ LoD 5 µg/kg; AFB1 = Aflatoxin B1; AFB2 = Aflatoxin B2; AFG1 = Aflatoxin G1; AFG2 = Aflatoxin G2; DON = Deoxynivalenol; FuB1 = Fumonisin B1; FuB2 = Fumonisin B2; HT2 = HT-2-toxin; NIV = Nivalenol; OTA = Ochratoxin A; T2 = T-2 toxin; ZEA = Zearalenone; MRP = malt residual pellets.

**Table 3:** Element analysis of various food and feed products.

Parameter	MRP ^1^ [mg/kg]	WB ^2^ [mg/kg]	Diet Feed LCR FARMAX [mg/kg]	Insect Meal [mg/kg]
Aluminum	61.6	5.3	26.1	1.6
Boron	3.7	4.0	6.5	2.1
Barium	10.3	7.3	8.2	2.7
Calcium	2168.3	730.0	8094.9	688.9
Cobalt	<0.2	<0.2	<0.2	<0.2
Copper	10.6	18.8	10.5	26.4
Iron	97.2	150.45	142.6	61.6
Potassium	15,218.2	15,752.1	10,894.3	6148.6
Magnesium	1635.5	6022.6	3180.5	2400.4
Manganese	44.8	145.3	127.2	14.6
Molybdenum	0.6	1.8	0.5	1.0
Sodium	333.0	41.1	864.1	1046.2
Nickel	0.3	1.3	0.5	0.4
Phosphorous	5069.0	16,752.0	8720.3	6936.7
Sulfur	2303.8	1961.2	3460.5	3865.3
Selenium	<0.2	<0.2	<0.2	0.3
Silicium	988.0	130.9	100.0	10.8
Strontium	6.5	3.7	9.6	2.4
Tin	<0.2	<0.2	<0.2	<0.2
Uranium	<0.2	<0.2	<0.2	<0.2
Vanadium	<0.2	<0.2	<0.2	<0.2
Zinc	63.4	133.7	84.1	182.5

^1^ Malt residue pellets; ^2^ Wheat bran.

**Table 4:** Analysis of heavy metals of various food and feed products.

Parameter	MRP ^1^ [mg/kg]	WB ^2^ [mg/kg]	Diet Feed LCR FARMAX [mg/kg]	Insect Meal [mg/kg]
Arsenic	<0.2	<0.2	<0.2	<0.2
Cadmium	<0.2	<0.2	<0.2	<0.2
Chromium	0.4	<0.2	<0.2	<0.2
Mercury	<0.2	<0.2	<0.2	<0.2
Lead	<0.2	<0.2	<0.2	<0.2
Antimony	<0.2	<0.2	<0.2	<0.2

^1^ Malt residue pellets; ^2^ Wheat bran.

## Data Availability

The raw data supporting the conclusions of this article will be made available by the authors on request.

## References

[1] Adler T. (2022). Insects on our Plates? Potential, Challenges and Opportunities in the Edible Insect Market. UCL J. Econ..

[2] Turck D., Bohn T., Castenmiller J., De Henauw S., Hirsch-Ernst K.I., Maciuk A., Mangelsdorf I., McArdle H.J., Naska A., EFSA Panel on Nutrition, Novel Foods and Food Allergens (NDA) (2021). Safety of frozen and dried formulations from whole yellow mealworm (*Tenebrio molitor larva*) as a novel food pursuant to Regulation (EU) 2015/2283. EFSA J..

[3] Pöllinger-Zierler B., Lienhard A., Mayer C., Berner S., Rehorska R., Schöpfer A., Grasser M. (2023). *Tenebrio molitor* (Linnaeus, 1758): Microbiological Screening of Feed for a Safe Food Choice. Foods.

[4] Truzzi C., Illuminati S., Girolametti F., Antonucci M., Scarponi G., Ruschioni S., Riolo P., Annibaldi A. (2019). Influence of Feeding Substrates on the Presence of Toxic Metals (Cd, Pb, Ni, As, Hg) in Larvae of *Tenebrio molitor*: Risk Assessment for Human Consumption. Int. J. Environ. Res. Public Health.

[5] Garofalo C., Osimani A., Milanović V., Taccari M., Cardinali F., Aquilanti L., Riolo P., Ruschioni S., Isidoro N., Clementi F. (2017). The microbiota of marketed processed edible insects as revealed by high-throughput sequencing. Food Microbiol..

[6] Ochoa Sanabria C., Hogan N., Madder K., Gillott C., Blakley B., Reaney M., Beattie A., Buchanan F. (2019). Yellow Mealworm Larvae (*Tenebrio molitor*) Fed Mycotoxin-Contaminated Wheat—A Possible Safe, Sustainable Protein Source for Animal Feed?. Toxins.

[7] Candian V., Scarpino V., Bona A., Tedeschi R., Blandino M. (2025). Mycotoxins-contaminated wheat matrices bioconversion by *Tenebrio molitor* larvae (Coleoptera: Tenebrionidae). Insect Sci..

[8] Niermans K., Woyzichovski J., Kröncke N., Benning R., Maul R. (2019). Feeding study for the mycotoxin zearalenone in yellow mealworm (*Tenebrio molitor*) larvae—Investigation of biological impact and metabolic conversion. Mycotoxin Res..

[9] Bakker M.G., Brown D.W., Kelly A.C., Kim H.-S., Kurtzman C.P., Mccormick S.P., O’Donnell K.L., Proctor R.H., Vaughan M.M., Ward T.J. (2018). *Fusarium* mycotoxins: A trans-disciplinary overview. Can. J. Plant Pathol..

[10] Rumbos C.I., Karapanagiotidis I.T., Mente E., Psofakis P., Athanassiou C.G. (2020). Evaluation of various commodities for the development of the yellow mealworm, *Tenebrio molitor*. Sci. Rep..

[11] Inácio J., Pereira P., Carvalho M., Fonseca Á., Amaral-Collaço M.T., Spencer-Martins I. (2002). Estimation and Diversity of Phylloplane Mycobiota on Selected Plants in a Mediterranean-Type Ecosystem in Portugal. Microb. Ecol..

[12] Essel E., Xie J., Deng C., Peng Z., Wang J., Shen J., Xie J., Coulter J.A., Li L. (2019). Bacterial and fungal diversity in rhizosphere and bulk soil under different long-term tillage and cereal/legume rotation. Soil Tillage Res..

[13] Into P., Pontes A., Sampaio J.P., Limtong S. (2020). Yeast Diversity Associated with the Phylloplane of Corn Plants Cultivated in Thailand. Microorganisms.

[14] Wang Q., Han Y., Yu Z., Tian S., Sun P., Shi Y., Peng C., Gu T., Li Z. (2025). Fungi in Horticultural Crops: Promotion, Pathogenicity and Monitoring. Agronomy.

[15] Bullerman L.B., Schroeder L.L., Park K.-Y. (1984). Formation and Control of Mycotoxins in Food. J. Food Prot..

[16] Iqbal S.Z. (2021). Mycotoxins in food, recent development in food analysis and future challenges; a review. Curr. Opin. Food Sci..

[17] Pinton P., Terciolo C., Payros D., Oswald I.P. (2025). Mycotoxins hazard: The European view. Curr. Opin. Food Sci..

[18] Pavicich M.A., De Boevre M., De Saeger S. (2025). Hidden mycotoxin risks in the shift toward plant-based diets. Curr. Opin. Food Sci..

[19] Meijer N., van Zadelhoff K., Dame-Korevaar M.A., Borghuis A., van Groenestijn J.W., Boonstra J., Brouwer M., Brust H., de Lange E., Leenders L. (2026). Food and feed safety of *Tenebrio molitor* reared on yet to be legally authorised residual streams in the European Union. J. Insects Food Feed.

[20] Mlček J., Adámek M., Adámková A., Matyáš J., Bučková M., Mrázková M., Vícha R., Vychodil R., Knížková I., Volek Z. (2021). Feed Parameters Influencing the Breeding of Mealworms (*Tenebrio molitor*). Sustainability.

[21] Napoli C., Marcotrigiano V., Montagna M.T. (2012). Air sampling procedures to evaluate microbial contamination: A comparison between active and passive methods in operating theatres. BMC Public Health.

[22] Ribeiro T., Pereira V. (2023). *Tenebrio molitor* powder: An analysis of the techniques for the production of powder at an industrial level. J. Insects Food Feed..

[23] (2017). Foodstuffs—Determination of Water Activity.

[24] (2020). Futtermittel: Probenahme-Und Untersuchungsverfahren_- Bestimmung von Deoxynivalenol, Aflatoxin B1, Fumonisin B1 Und B2, T-2- Und HT-2-Toxine, Zearalenon Und Ochratoxin_A in Einzelfuttermitteln Und Mischfuttermitteln Mittels LC-MS/MS.

[25] Cuero R.G., Smith J.E., Lacey J. (1987). Interaction of water activity, temperature and substrate on mycotoxin production by *Aspergillus flavus, Penicillium viridicatum* and *Fusarium graminearum* in irradiated grains. Trans. Br. Mycol. Soc..

[26] (2009). Wasserbeschaffenheit—Bestimmung von Ausgewählten Elementen Durch Induktiv Gekoppelte Plasma-Atom-Emissionsspektrometrie (ICP-OES).

[27] (2024). Wasserbeschaffenheit—Anwendung Der Induktiv Gekoppelten Plasma-Massenspektrometrie (ICP-MS)—Teil 2: Bestimmung von Ausgewählten Elementen Einschließlich Uran-Isotope.

[28] Vandeweyer D., Crauwels S., Lievens B., Van Campenhout L. (2017). Microbial counts of mealworm larvae (*Tenebrio molitor*) and crickets (*Acheta domesticus* and *Gryllodes sigillatus*) from different rearing companies and different production batches. Int. J. Food Microbiol..

[29] Londoño-Hernández L., Ramírez-Toro C., Ruiz H.A., Ascacio-Valdés J.A., Aguilar-Gonzalez M.A., Rodríguez-Herrera R., Aguilar C.N. (2017). Rhizopus oryzae—Ancient microbial resource with importance in modern food industry. Int. J. Food Microbiol..

[30] Ghosh B., Ray R. (2011). Current Commercial Perspective of Rhizopus oryzae: A Review. J. Appl. Sci..

[31] Raiesi O., Hashemi S.J., Getso M.I., Ardi P., Mohammadi Ardehali M., Raissi V., Shamsaei S., Borjian Boroujeni Z. (2021). First report of chronic invasive fungal rhinosinusitis in a patient with ovarian cancer caused by Didymella pedeiae and successful treatment with voriconazole: A case report. Curr. Med. Mycol..

[32] Ribeiro J.M.M., Cavaglieri L.R., Fraga M.E., Direito G.M., Dalcero A.M., Rosa C.A.R. (2006). Influence of water activity, temperature and time on mycotoxins production on barley rootlets. Lett. Appl. Microbiol..

[33] Sautour M., Soares Mansur C., Divies C., Bensoussan M., Dantigny P. (2002). Comparison of the effects of temperature and water activity on growth rate of food spoilage moulds. J. Ind. Microbiol. Biotechnol..

[34] Ruiz-Medina A., Fernández-de Córdova M.L. (2015). Aflatoxin B1 in Beer at Different Stages of Production. Processing and Impact on Active Components in Food.

[35] Council of the European Union, European Parliament (2002). Directive 2002/32/EC of the European Parliament and of the Council of 7 May 2002 on Undesirable Substances in Animal Feed—Council Statement.

[36] Council of the European Union, European Parliament (2006). Commission Recommendation of 17 August 2006 on the Presence of Deoxynivalenol, Zearalenone, Ochratoxin A, T-2 and HT-2 and Fumonisins in Products Intended for Animal Feeding (Text with EEA Relevance).

[37] van der Fels-Klerx H.J., Camenzuli L., van der Lee M.K., Oonincx D.G.A.B. (2016). Uptake of Cadmium, Lead and Arsenic by *Tenebrio molitor* and *Hermetia illucens* from Contaminated Substrates. PLoS ONE.

[38] Mirzaeva D.A., Khujamshukurov N.A., Zokirov B., Soxibov B.O., Kuchkarova D.K. (2020). Influence of Temperature and Humidity on the Development of *Tenebrio molitor* L. Int. J. Curr. Microbiol. Appl. Sci..

[39] Marín P., Magan N., Vázquez C., González-Jaén M.T. (2010). Differential effect of environmental conditions on the growth and regulation of the fumonisin biosynthetic gene FUM1 in the maize pathogens and fumonisin producers Fusarium verticillioides and Fusarium proliferatum: Ecophysiology of *F. verticillioides* and *F. proliferatum*. FEMS Microbiol. Ecol..

[40] Hagiuda R., Hirose D. (2024). Species diversity of xerophilic *Aspergillus* and *Penicillium* in marine surface waters revealed by isolation using osmophilic medium. J. Microorg. Control.

[41] Coleine C., Stajich J.E., Selbmann L. (2022). Fungi are key players in extreme ecosystems. Trends Ecol. Evol..

[42] Stevenson A., Hamill P.G., O’Kane C.J., Kminek G., Rummel J.D., Voytek M.A., Dijksterhuis J., Hallsworth J.E. (2017). Aspergillus penicillioides differentiation and cell division at 0.585 water activity. Environ. Microbiol..

[43] Dallyn H., Everton J.R. (1969). The xerophilic mould, *Xeromyces bisporus*, as a spoilage organism. Int. J. Food Sci. Technol..

[44] Pitt J.I., Christian J.H.B. (1968). Water Relations of Xerophilic Fungi Isolated from Prunes. Appl. Microbiol..

[45] Escott C., Fresno J.M.D., Loira I., Morata A., Suárez-Lepe J.A. (2018). *Zygosaccharomyces rouxii*: Control Strategies and Applications in Food and Winemaking. Fermentation.

[46] Wang J., Xie Z., Feng Y., Huang M., Zhao M. (2024). Co-culture of *Zygosaccharomyces rouxii* and *Wickerhamiella versatilis* to improve soy sauce flavor and quality. Food Control.

[47] Mateos C.J., Aguilar M.V., Martínez-Para M.C. (2003). Determination of the Chromium Content in Commercial Breakfast Cereals in Spain. J. Agric. Food Chem..

[48] Rasool A., Pertile E., Brožová K., Halfar J., Čabanová K., Malíková P., Chromíková J., Motyka O., Drabinová S., Heviánková S. (2025). Mechanistic insights and environmental ramifications of Cr (III) oxidation to Cr (VI) in soil and groundwater systems: Bridging geochemical mechanisms and emerging remediation strategies. Environ. Geochem. Health.

[49] Sykuła-Zając A., Pawlak A. (2012). Chromium in food products. Biotechnol. Food Sci..

[50] Farri R., Gimino M.J., Lagarda M.J. (1987). The occurrence of chromium in raw materials and its fate in the brewing process. J. Inst. Brew..

[51] Malematja E., Manyelo T.G., Sebola N.A., Kolobe S.D., Mabelebele M. (2023). The accumulation of heavy metals in feeder insects and their impact on animal production. Sci. Total Environ..

